# Reperfusion injury on computed tomography following endovascular revascularization of acute mesenteric ischemia: prevalence, risk factors, and patient outcome

**DOI:** 10.1186/s13244-022-01339-9

**Published:** 2022-12-13

**Authors:** Lorenzo Garzelli, Alexandre Nuzzo, Annick Hamon, Iannis Ben Abdallah, Jules Gregory, Lucas Raynaud, Luisa Paulatto, Marco Dioguardi Burgio, Yves Castier, Yves Panis, Valérie Vilgrain, Olivier Corcos, Maxime Ronot

**Affiliations:** 1 Université Paris Cité, Paris, France; 2 Service de Radiologie, Hôpital Beaujon, APHP.Nord, 100 Blvd du Général Leclerc, 92118 Clichy, France; 3 Intestinal Stroke Center, Service de Gastroenterology, MICI Et Insuffisance Intestinale, Hôpital Beaujon, APHP.Nord, Clichy, France; 4 Service de Chirurgie Vasculaire, Hôpital Bichat, APHP.Nord, Paris, France; 5 Service de Chirurgie Digestive, Hôpital Bichat, APHP.Nord, Paris, France

**Keywords:** Endovascular revascularization, Mesenteric ischemia, Stroke center, Small bowel

## Abstract

**Background:**

Data about reperfusion injury (RI) following acute arterial mesenteric ischemia (AAMI) in humans are scarce. We aimed to assess the prevalence and risk factors of RI following endovascular revascularization of AMI and evaluate its impact on patient outcomes.

**Methods:**

Patients with AAMI who underwent endovascular revascularization (2016–2021) were included in this retrospective cohort. CT performed < 7 days after treatment was reviewed to identify features of RI (bowel wall hypoattenuation, mucosal hyperenhancement). Clinical, laboratory, imaging, and treatments were compared between RI and non-RI patients to identify factors associated with RI. Resection rate and survival were also compared.

**Results:**

Fifty patients (23 men, median 72-yrs [IQR 60–77]) were included, and 22 were diagnosed with RI (44%) after a median 28 h (22–48). Bowel wall hypoattenuation and mucosal hyperenhancement were found in 95% and 91% of patients with post-interventional RI, respectively. Patients with RI had a greater increase of CRP levels after endovascular treatment (*p* = 0.01). On multivariate analysis, a decreased bowel wall enhancement on baseline CT (HR = 8.2), an embolic cause (HR = 7.4), complete SMA occlusion (HR = 7.0), and higher serum lactate levels (HR = 1.4) were associated with RI. The three-month survival rate was 78%, with no difference between subgroups (*p* = 0.99). However, the resection rate was higher in patients with RI (32% versus 7%; *p* = 0.03).

**Conclusion:**

RI is frequent after endovascular revascularization of AAMI, especially in patients who present with decreased bowel wall enhancement on pre-treatment CT, an embolic cause, and a complete occlusion of the SMA. However, its occurrence does not seem to negatively impact short-term survival.

**Supplementary Information:**

The online version contains supplementary material available at 10.1186/s13244-022-01339-9.

## Key points

- Reperfusion injury of the bowel occurred in 44% of patients with acute mesenteric ischemia treated by endovascular revascularization.
- A decreased bowel enhancement on initial CT (HR = 8.2), an occlusion of the superior mesenteric artery (HR = 7.0), an embolic cause (HR = 7.4), and higher initial serum lactate level (HR = 1.4) were identified as predictors of reperfusion injury.
- Bowel wall hypoattenuation, hyperenhancement of the mucosa, and increase in CRP levels after revascularization were key diagnostic features of reperfusion injury.

**Supplementary Information:**

The online version contains supplementary material available at 10.1186/s13244-022-01339-9.

## Introduction

Acute mesenteric ischemia is defined by the association of mesenteric vascular insufficiency (that can be occlusive or non-occlusive) with ischemic gut injury (that can be reversible or irreversible when transmural necrosis occurs). Arterial acute mesenteric ischemia (AAMI) is a life-threatening condition, associated with a dismal prognosis: nearly 100% mortality rate without appropriated bowel resection or revascularization [1] and 27% to 63% when revascularization is attempted [1–6]. Endovascular revascularization in AAMI is one of the cornerstones of the treatment to save or limit the bowel damage [7, 8]. Depending on the mechanism (atherothrombosis or emboli), the localization, and the extent of superior mesenteric artery (SMA) occlusion, endovascular techniques include intra-arterial thrombolysis [9–11], thrombus-aspiration [12–14], or stenting [2, 15].

Ischemia–reperfusion injury (RI) following mesenteric ischemia is caused by restoring blood flow in a damaged bowel leading to additional cell injury [16]. The underlying mechanism of RI is not specific to the bowel and is a result of the oxidative stress following reperfusion that increases reactive oxygen species, and local inflammation leading to cell death [17]. This complex and multifactorial pathophysiological process is not yet fully understood and has been studied in preclinical models in various organs especially the heart, the brain, the liver, and the bowel [17, 18].

Abundant research in animal models has focused on histological [19, 20] and absorptive changes in the bowel [21] or bacterial translocation caused by mucosal disruption [22]. The few studies evaluating the macroscopic findings of RI in animals or human surgical models have shown that lesions lead to intra-luminal and bowel wall hemorrhage [23, 24].

Preclinical imaging studies using computed tomography (CT) or magnetic resonance imaging (MRI) to assess RI are scarce [25–28]. In clinical research, RI has been described as a component of ischemic colitis [29, 30], in non-occlusive mesenteric ischemia [31], or in a case report following endovascular revascularization of severe chronic mesenteric ischemia [32]. All consistently report a thickening of the bowel wall due to submucosal edema and/or hemorrhage with adjacent fat stranding and ascites either after revascularization [32] or spontaneously in non-occlusive mesenteric ischemia [31] or colon infarction [29]. In contrast to the extensive literature of preclinical studies of bowel RI [33], no clinical research has been published on RI in AAMI.

Thus, the purpose of this study was to assess the prevalence and risk factors of RI in a cohort of AAMI patients treated by endovascular revascularization and evaluate its impact on patient outcomes.

## Material and methods

### Patient population

The Institutional Review Board approved this single-center retrospective observational study (CRM-2205-266), performed according to the ethical standards of the Committee on Human Experimentation of our institution and reported according to the Strengthening the Reporting of Observational Studies in Epidemiology (STROBE) guidelines.

All patients with AAMI that were prospectively admitted to the intestinal stroke center unit of Beaujon Hospital (SURVI unit, Clichy, France) were considered. Inclusion criteria were: (i) patients diagnosed with arterial occlusive mesenteric ischemia on contrast-enhanced CT and (ii) who were treated by endovascular revascularization. Exclusion criteria were as follows: (i) patients who underwent surgery (any laparotomy or laparoscopy) before undergoing follow-up CT after endovascular revascularization, (ii) patients without CT within seven days from the endovascular revascularization, and (iii) presenting with bowel thickening on CT before revascularization. We chose to exclude patients who underwent laparotomy for open revascularization or bowel resection because surgery could influence the appearance of the bowel. We hypothesized that RI could resolve in patients evaluated by CT more than 7 days after revascularization and therefore those patients were excluded. Patients with bowel thickening before revascularization could have RI caused by spontaneous clot dissolution and were also excluded. Patient history, clinical, laboratory (serum lactate, white blood cell (WBC), creatinine, C-reactive protein (CRP)), and pathological data were prospectively obtained, as well as additional treatments and patient outcomes. CTs were retrospectively reviewed by two abdominal radiologists in consensus (M.R. and L.G. with 12 and 3 years of experience) who were unaware of the final diagnosis of RI or not.

### Diagnosis of acute mesenteric ischemia

In our cohort, the diagnosis of AAMI was based on an association of (a) acute intestinal symptoms (e.g., intense and sudden pain, diarrhea), (b) arterial involvement on CT (severe stenosis or occlusion of the superior mesenteric artery), (c) related bowel abnormalities consistent with ischemic injury on CT (decreased enhancement, pneumatosis and/or bowel dilatation), and d) the absence of alternative cause. It should be noted that bowel abnormalities could be missing in very early forms of this disease. In these cases, a diagnosis of AAMI was considered if the three other criteria were all met. The final diagnosis was determined by an on-call group of AAMI experts, including gastroenterologists, surgeons, and radiologists. SMA lesions were retrospectively graded according to Society of Cardiovascular Computed Tomography guidelines for the interpretation and reporting of CT coronary angiography [34] as minimal (< 25%), mild (25%-49%), moderate (50%-69%), severe (70–99%), or occlusion. Lesions were also retrospectively classified as proximal (between the ostium and the origin of the inferior pancreaticoduodenal artery), middle (between the origin of the first jejunal artery and the origin of the ileocolic artery), and distal (after the origin of the ileocolic artery). Lesions of the coeliac trunk were also reported and graded. Arterial embolus was considered to be the etiology when other infarcts were observed or in case of no underlying atherosclerosis. CT features of AAMI such as decreased enhancement, bowel loop dilatation, or pneumatosis were also reported.

### Treatment strategy

All admitted patients were treated according to the institutional treatment protocol established by Corcos et al. [35]. After admission, all patients received the multimodal medical protocol including oral antibiotic coverage [36], anti-platelet and anticoagulation therapy, bowel resting, and blood volume resuscitation.

When considered to be technically possible (patent femoral or brachial access, no severe atherosclerosis such as coral reef aorta), endovascular revascularization was systematically proposed as an upfront treatment except in patients with an initial high probability of extensive intestinal necrosis assessed by the Clichy score (that stratifies the probability of transmural necrosis based on three items: serum lactate > 2 mmol/l, bowel loop dilatation > 25 mm, and presence of an organ failure) [37].

### Endovascular revascularization

Endovascular revascularization was either performed by an interventional radiologist or a vascular surgeon depending on the availability of each team at the time of the diagnosis. Endovascular revascularization methods included stenting, thrombus-aspiration and/or in situ thrombolysis. The choice of method was decided considering the first SMA angiogram and the pre-treatment CT. Either manual (with a 50-mL syringe) or mechanical (penumbra aspiration pump with a dedicated catheter; Indigo Catheter, Penumbra, Inc., Alameda, CA) thrombus-aspiration was typically performed for clots in the medial part of the SMA. When complete recanalization of all branches was not obtained, transluminal angioplasty with stenting (covered or bare-metal) or intra-arterial thrombolysis (bolus of 8 mg of alteplase [Actilyse, Boehringer Ingelheim], or 100,000 IU of urokinase [Actosolv, Eumedica] followed by 1 mg/h of alteplase or 100,000 UI/hour delivered with a syringe pump) could be used in adjunction to the thrombus-aspiration, depending on the localization of the residual occlusion/stenosis. Stenting was preferred for proximal lesions (embolic or atherothrombotic), and thrombolysis was attempted in case of distal clots (i.e., not accessible to thrombus-aspiration). Technical success was defined as successful arterial recanalization during completion angiogram with no residual significant stenosis (partial or total recanalization). This definition is arbitrary as there is no consensus or recommendation on technical success of endovascular revascularization in AAMI as opposed to cerebral (thrombolysis in cerebral infarction score) or myocardial (blush grade) revascularization. Technical failure was defined as no improvement after the recanalization attempt. In patients who underwent thrombolysis, the completion angiogram was the one obtained at the end of treatment, i.e., when the intra-arterial thrombolysis catheter was removed.

If the endovascular treatment failed technically or did not improve the clinical status, laboratory tests (e.g., serum lactate), or follow-up imaging, patients were referred for open revascularization and surgical bowel resection, when needed. Perioperative complications of endovascular revascularization were recorded according to the Society of Interventional Radiology guidelines [38] but not systematically searched for. Vascular patency at the first control CT was also evaluated.

### Reperfusion injury

A follow-up CT was performed in our institution within 7 days after revascularization even in the absence of clinical symptoms or biological abnormalities to assess the result of the treatment on the bowel and the splanchnic vasculature. A CT was also performed in patients with clinical symptoms or biological abnormalities to search for possible AAMI recurrence or complication. (The CT protocol is detailed in Additional file 1.) RI was defined as the appearance of a thickened bowel segment (arbitrarily assessed as a bowel wall > 5 mm) in association with at least one of the following findings: hyperenhancement of the mucosa, edema of the submucosa (defined as a marked hypoattenuation on portal venous phase), and fat stranding or fluid adjacent to the thickened bowel loop segment [39]. Venous dilatation in the meso of the involved bowel loop was also visually detected and reported, as well as the presence of a luminal hemorrhage on unenhanced CT. The delay between the initial CT and revascularization and between revascularization and the follow-up CT, when RI was documented, was retrieved, together with the indication for CT. New abdominal pain, bloody diarrhea, or changes in serum lactate, CRP, and WBC after revascularization and before the follow-up CT were noted and compared between patients with or without RI.

Resection rate, three-month survival, and intra-hospital death rates were reported. In patients with RI, a follow-up CT at one year was performed to evaluated bowel abnormality (e.g., bowel thickening) persistence and survival.

### Statistical analysis

Results are presented as medians (interquartile range), means ± standard deviations (SD), or the number of cases (percentage of cases). Patients with and without reperfusion injury were compared with the Student’s t test or the Mann–Whitney test for continuous variables and the Fisher exact test for categorical variables. Survival was calculated as the delay between the date of revascularization and death by any cause. The survival curves were presented using the Kaplan–Meier method and compared using the log rank. Tests were two-sided, and *p* < 0.05 was considered to be significant. Variables associated with the occurrence of reperfusion injury in univariate analysis (*p* < 0.05) were entered in a Cox proportional hazards regression model. Two models were computed: a classic “all-in-one” model including all significant variables on univariate analysis, and a two-step chronological model considering pre-revascularization characteristics, first, then treatment-related variables. Independent variables were used to compute a predictive score for the occurrence of RI, and confidence intervals for the receiver operating characteristics curve were calculated. All analyses were performed using Prism version 9.0.0 (GraphPad Software, San Diego, California, USA) and the Statistical Package for the Social Sciences (SPSS) (version 26.0, SPSS Inc., Chicago, IL, USA).

## Results

### Population

**Fig. 1:**
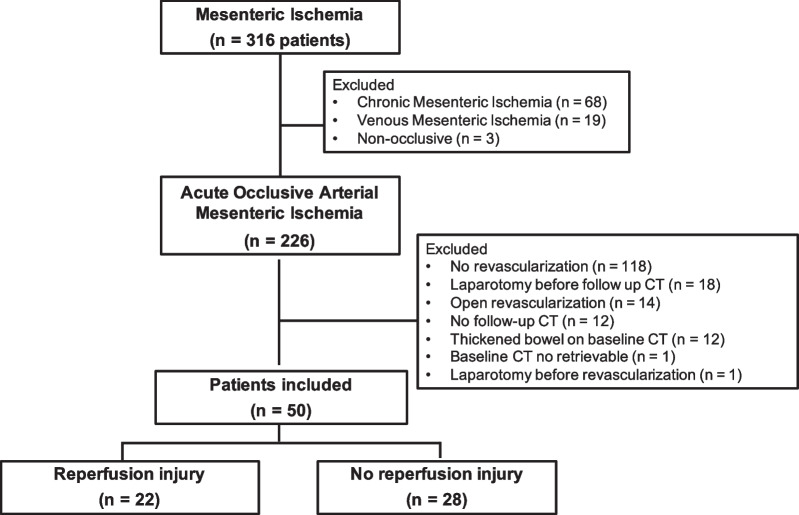
Flowchart of the study

From January 2016 to October 2021, 316 patients who were admitted for AAMI were screened. Two hundred and twenty-six (71%) had an AAMI. After applying the exclusion criteria, 50 patients (27 women, 54%) were included. (Flowchart is provided in Fig. 1.) Patient characteristics are provided in Table 1.

**Table 1:** Baseline characteristics

	All patients *n* = 50	No Reperfusion Injury *n* = 28 (56%)	Reperfusion Injury *n* = 22 (44%)	*p* value
Women	27 (55%)	11 (39%)	16 (73%)	**0.02**
Age at revascularization (years)	72 (60–77)	75 (68–77)	73 (57–82)	0.93
Median delay Initial CT—Revascularization (hours)*	7 (5–22)	18 (6–51)	6 (5–10)	** < 0.01**
Serum lactate, (mmol/L)*	1.8 (1.4–2.3)	1.6 (1.0–2.1)	1.9 (1.1–2.6)	**0.02**
WBC†, (G/L)	13 (9.8–18.3)	11.3 (9–18)	15.2 (12.8–19.4)	**0.02**
CRP‡, mg/L	46 (17–94)	77 (15–156)	31 (17–62)	0.13
Clichy Score*				0.05
* 0–1*	36 (73%)	23 (85%)	14 (59%)	
* 2–3*	13 (27%)	4 (15%)	9 (41%)	
CT Features				
SMA Occlusion	30 (60%)	13 (46%)	17 (77%)	**0.04**
Site of predominant vascular Lesion				** < 0.01**
* Ostial/Proximal SMA*	17 (35%)	15 (56%)	2 (9%)	
* Middle/Distal SMA*	33 (66%)	13 (46%)	20 (91%)	
Celiac Trunk stenosis > 70%	6 (12%)	4 (14%)	2 (9%)	0.68
Bowel dilatation	16 (32%)	9 (32%)	8 (36%)	0.77
Decreased enhancement	20 (41%)	3 (11%)	17 (77%)	** < 0.01**
Bowel wall pneumatosis	0 (-)	0 (-)	0 (-)	–
Etiology				** < 0.01**
* Emboli*	25 (50%)	8 (29%)	17 (77%)	
* Thrombosis*	25 (50%)	20 (71%)	5 (23%)	

Results are presented as medians and interquartile ranges

*Missing data for one patient. †Missing data in 3 patients. ‡Missing date in 6 patients.

*CRP* (C-reactive protein); *CT* (computed tomography); *SMA* (superior mesenteric artery); and *WBC* (white blood cells)

Bold values indicate statistical significant values

### Revascularization

Retrospective evaluation of the SMA before revascularization found an occlusion in 30/50 patients (60%), a > 70% stenosis in four patients (8%), a 50 to 70% stenosis in 11 patients (22%), a 25% to 50% of stenosis in three patients (6%), and a < 25% stenosis in two patients (4%).

The technical success rate was 92% (46/50 patients). Stenting was used in 21/50 patients (42%), intra-arterial thrombolysis in 24/50 patients (48%), and thrombus-aspiration in 20/50 patients (40%). Fifteen patients (30%) were treated with a combination of techniques (Table 2). The four patients with a technical failure received thrombolysis. One of them died from extensive bowel necrosis (3 days after the revascularization), one underwent open revascularization and died from extensive necrosis (1 day after endovascular revascularization), and two underwent resection of necrotic segments of the bowel.

**Table 2:** Revascularization details and outcomes

	All patients (*n* = 50)	No reperfusion Injury (*n* = 28)	Reperfusion injury (*n* = 22)	*p* value
Endovascular revascularization				
Techniques				
* Thrombolysis*	24 (49%)	9 (33%)	15 (68%)	**0.02**
* Stenting*	21 (42%)	16 (57%)	5 (23%)	**0.01**
* Thrombus-aspiration*	20 (40%)	6 (21%)	14 (63%)	** < 0.01**
≥ *2 combined techniques*	18 (36%)	6 (21%)	12 (54%)	**0.02**
Technical Failure	4 (8%)	2 (7%)	2 (9%)§	0.99
Adverse event				0.07
* Absence*	39 (80%)	21 (78%)	18 (82%)	
* Mild/Moderate*	5 (10%)	5 (18%)	0 (-)	
* Severe*	6 (12%)	2 (7%)	4 (18%)	
Follow-up CT				
Delay revascularization-CT (hours)¤	39 (18–65)	42 (15–71)	28 (20–48)	0.39
Indication of CT				
*Systematic follow-up*	24 (48%)	13 (46%)	11 (50%)	
*Abdominal pain*	13 (26%)	9 (32%)	4 (18%)	
*Clinical worsening*	3 (6%)	1 (4%)	2 (9%)	
*Inflammatory syndrome*	3 (6%)	2 (7%)	1 (5%)	
*Elevated serum lactate*	3 (6%)	1 (4%)	2 (9%)	
*Internal bleeding suspicion*	4 (8%)	2 (7%)	2 (9%)	
SMA Recanalization				0.49
* None*	8 (16%)	6 (21%)	2 (9%)¥	
* Partial*	19 (38%)	10 (36%)	9 (41%)	
* Complete*	23 (46%)	12 (43%)	11 (50%)	
Clinical/Biological change after revascularization				
Abdominal pain†	26 (55%)	11 (42%)	15 (71%)	0.07
Bloody diarrhea‡	8 (17%)	3 (12%)	5 (25%)	0.26
Change in serum lactate (%)‡	− 5% (− 38%; + 73%)	0% (− 33%; + 95%)	− 6% (− 43%; + 68%)	0.42
Change in WBC (%)‡	− 6% (− 25%, + 13%)	− 5% (− 19%, + 15%)	− 9% (− 32%; + 10%)	0.45
Change in CRP (%)*	+ 93% (− 6%; + 408%)	+ 5% (− 36%, + 171%)	+ 371% (+ 100; + 495%)	** < 0.01**
Survival and morbidity				
Secondary bowel resection	9 (18%)	2 (7%)	7 (32%)	**0.03**
Mean resected bowel length (cm)	80 (46–100)	48 (16–80)	80 (46–115)	0.33
3-month survival	38 (76%)	22 (79%)	16 (73%)	0.74

Results are presented as medians and interquartile ranges

All twelve deaths were in-hospital

^*^Missing data in 16 patients

^†^Missing data in 3 patients

^‡^Missing date in 4 patients

¤Missing data in one patient

¥One patient had partial revascularization of the SMA on the final angiogram, and the other was treated by intra-arterial thrombolysis

^§^Those two patients were treated by intra-arterial thrombolysis

CRP (C-reactive protein); CT (computed tomography); and WBC (white blood cells)

Bold values indicate statistical significant values

Severe complications occurred in 6/50 patients (12%), all related to the femoral access (hematoma (*n* = 2), false aneurysms (*n* = 3), or hemorrhagic shock (*n* = 1, that occurred in the intensive care unit a few hours after revascularization). Two patients had mild complications: One procedure was complicated with the appearance of a small distal clot at the end of the intervention without clinical consequences, and in another one, a small SMA ileal branch was perforated during catheterization with contrast extravasation but was spontaneously resolutive.

Revascularization was more frequently performed in patients with RI by thrombus-aspiration (*p* < 0.01) and intra-arterial thrombolysis (*p* = 0.02) (Table 2).

### Reperfusion injury on CT

Follow-up CT was performed a median of 39 h (18–65) after revascularization. The reasons for performing follow-up CT were: systematic follow-up (*n* = 24; 48%), abdominal pain (*n* = 12; 24%), elevated serum lactate levels (*n* = 4; 8%), hemoglobin drop (*n* = 4; 8%), inflammatory syndrome (*n* = 3; 6%), and clinical aggravation (*n* = 3; 6%). On follow-up CT, complete and partial recanalization of the SMA was obtained in 23/50 (46%) and 19/50 (38%) patients, respectively (Table 2).

**Fig. 2:**
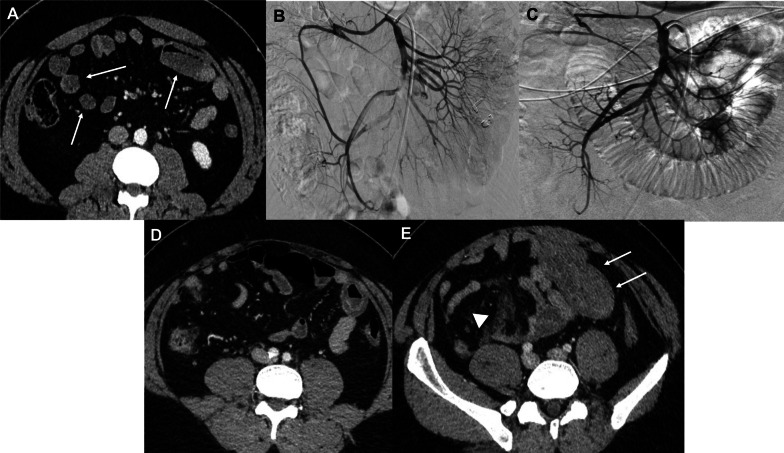
A 25-year-old male patient presented with early acute mesenteric ischemia (embolus of the superior mesenteric artery, SMA). Decrease in enhancement of the small bowel (arrow) compared to non-ischemic ones (arrowheads) on baseline CT portal-venous phase (**a**) with complete occlusion of the distal branches of the SMA (**b**). Complete recanalization of the SMA by thrombus-aspiration and in situ thrombolysis (**c**). Contrast-enhanced CT (portal venous phase) performed 12 h after revascularization for systematic post-procedural evaluation showed a thickened jejunal loop (arrow) with hyperenhancement of the mucosa and edema of the submucosa consistent with reperfusion injury (**d**). 24 h after revascularization, the patient has bloody diarrhea with anemia. On portal-venous phase CT, the previously thickened small bowel segment showed marked thickening with intra-luminal hyperattenuating material consistent with hemorrhage (arrow) and mesenteric fat stranding (arrowhead) (**e**). The patient was closely monitored surveillance, and no laparotomy was needed

**Fig. 3.**
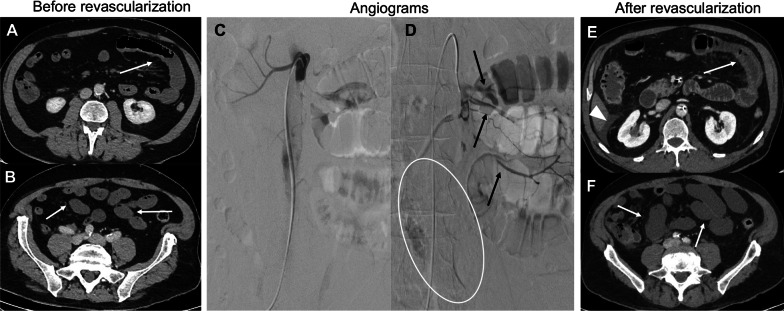
A 61-year-old male patient presenting with early acute mesenteric ischemia. The contrast-enhanced CT (portal venous phase) showed a decreased enhancement of the proximal jejunum (**a**) and ileum (**b**) wall. Digital subtraction angiography showed complete occlusion of the superior mesenteric artery (SMA) (**c**). Partial revascularization of the proximal jejunum arteries was achieved after 12 h of in situ thrombolysis with no opacification of the distal SMA (white oval) (**d**). Unfortunately, the patient experienced further clinical deterioration. Contrast-enhanced CT showed thickening of the jejunum wall with fat stranding (arrow) consistent with reperfusion injury following the recanalization of the jejunal arteries (**e**). Peri-hepatic fluid (arrowhead) was also noted. Additionally, CT showed a dilated jejunum with a persistent absence of wall enhancement, suggesting transmural necrosis of non-revascularized segments (**f**). The patient underwent a laparotomy that confirmed the absence of necrosis of the jejunum. He underwent subsequent resection of 225 cm of the small bowel and suffered from short bowel syndrome

Reperfusion injury was identified in 22 of the 50 patients (44%) on the CTs performed a median of 28 h (22–48) after revascularization. Mean bowel wall thickening in the bowel segment with RI was 8.5 mm (7–9). Bowel wall edema was the most frequent co-finding (21/22 patients; 95%). Hyperenhancement of the mucosa, fat stranding, and fluid adjacent to the involved bowel loop was found in (20/22 patients (91%), 20/22 patients (91%), and 18/22 patients (82%)), respectively. Draining veins were dilated in 20/22 patients (91%) confirming venous turgescence. Intra-luminal hemorrhage was found in 4/22 patients (18%). The jejunum was involved in 11/22 (50%) patients, the ileum in 16/22 (73%), the right colon in 3/22 (14%), and the left colon in 4/22 patients (18%). Nine patients (41%) had more than one bowel segment involved (Figs. 2 and 3).

### Univariate analysis of factors associated with reperfusion injury

Patients who developed RI were more frequently women (*p* = 0.02), with higher initial serum lactate levels (*p* = 0.02) and WBC (*p* = 0.02). There was a statistical trend associating a higher Clichy score (2 or 3/3) with RI (*p* = 0.05). No significant difference was found in terms of age at revascularization (*p* = 0.93), or initial CRP level (*p* = 0,13). The median delay between the initial CT and revascularization was longer in patients without a RI (*p* < 0.01).

On CT, the cause of AAMI was more frequently arterial emboli in RI patients (*p* < 0.01), and they had a higher rate of complete SMA occlusion (*p* < 0.01) with lesions located in the middle or distal part of the SMA (*p* < 0.01). Seventeen patients with RI initially had a decreased bowel wall enhancement (77%) of the bowel segment displaying RI, compared to three patients (11%) without RI (*p* < 0.01). No significant difference was found in terms of celiac trunk stenosis (*p* = 0.68) or bowel dilatation (*p* = 0.77).

The occurrence of abdominal pain (15 vs. 11 patients, *p* = 0.07) or bloody diarrhea (5 vs. 3 patients, *p* = 0.26) after revascularization was not significantly different between patients with and without RI (Table 2). Four patients had both pain and bloody diarrhea, and six had neither. The median WBC levels after revascularization in RI patients were 15 G/L (12–20) vs. 10 G/L (8–15) in those without RI (*p* = 0.02). The median CRP was 146 mg/L (110–204) after revascularization in RI patients vs. 62 mg/L (21–129) in patients without (*p* < 0.01). The median CRP increased by 371% (100%–495%) after revascularization in patients with RI versus 5% ( − 36%; 171%) in patients without RI (*p* < 0.01). There was no difference in median lactate change after revascularization: − 6% ( − 43%; + 68%) in RI patients versus 0% ( − 33%; + 95%) in non-RI patients (*p* = 0.42).

### Multivariate analysis of factors associated with reperfusion injury

The baseline clinical, laboratory, imaging data, and procedural data that were significantly associated with the occurrence of a RI on univariate analysis were used to perform a multivariate Cox proportional hazards regression model. When considering all variables, decreased bowel wall enhancement on pre-treatment CT (hazard ratio [HR] = 8.2; IC95% 2.0–33.0), an embolic cause (HR = 7.4; IC95% 1.5–34.7), complete occlusion of the SMA (HR = 7.0; IC95% 1.0–45.5), and higher initial lactate levels (HR = 1.4; IC95% 1.0–2.1) were independent predictors of the occurrence of RI (Table 3).

**Table 3:** Multivariate analysis for the identification of factors associated with reperfusion injury following endovascular revascularization, using a Cox proportional hazards regression model

Variables	HR	95%CI	*p* value
Women			0.863
Pre-treatment serum lactate (mmol/L)	1.47	1.02–2.11	0.034
Pre-treatment WBC (G/L)			0.670
Decreased bowel wall enhancement on pre-treatment CT	8.28	2.07–33.09	0.003
Complete SMA occlusion	7.04	1.09–45.53	0.04
Predominant vascular lesion in middle/distal SMA			0.168
Arterial emboli cause	7.42	1.58–34.72	0.011
Delay initial CT—revascularization (hours)			0.272
Treatment with thrombolysis			0.663
Treatment with stenting			0.297
Treatment with thrombus-aspiration			0.153

*CI* confidence interval; *CT* (computed tomography); *HR* (hazard ratio); *HR* hazard ratio; *SMA* (superior mesenteric artery); and *WBC* (white blood cells)

We then fit a two-step chronological model with the first pre-treatment variables (i.e., sex, serum lactate, WBC, arterial emboli cause, decreased enhancement of the bowel wall, degree of vascular occlusion, and location of the occlusion localization) in the first step and procedure-related variables (i.e., delay CT-revascularization, use of thrombus-aspiration, thrombolysis, and stenting) in the second step. The same variables were independently associated with the occurrence of RI: decreased bowel wall enhancement (HR = 8.5; IC95% 2.1–34.0), an emboli cause (HR = 7.4; IC95% 1.6–34.1), complete occlusion of the SMA (HR = 6.5; IC95% 1.0–41.9), and higher initial lactate levels (HR = 1.4; IC95% 1.0–2.0).

**Fig. 4:**
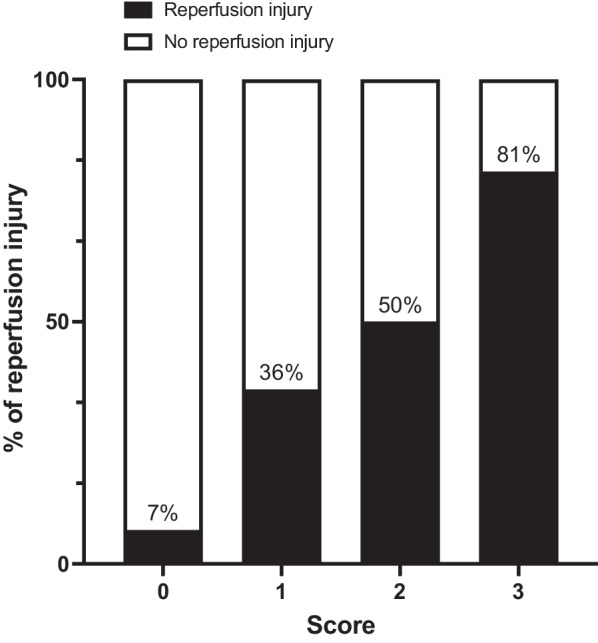
Rate of reperfusion injury after revascularization according to the computed tomography score. The score includes the presence of a decreased enhancement of the bowel wall on pre-treatment CT, an embolic as the cause of the mesenteric ischemia, and complete occlusion of the superior mesenteric artery (each equally graded as 1 point)

Based on these findings and after removal of the variable of lactate because of its lower weight, the rate of RI in the study population increased from 7% in patients with no predictive factors to 36%, 50%, and 81% in patients with 1, 2, and 3 factors, respectively (Fig. 4). This CT score had an area under the receiver operating characteristics curve to predict RI of 0.834 (95% confidence interval: 0.720–0.948). Addition of serum lactate did not improve the predictive value (AUC 0.832, 95% confidence interval: 0.714–0.951).

### Patient outcomes

Secondary bowel resection was required in 9/50 patients (18%), with a greater proportion in patients with RI (7/22 patients; 32%) than in those without (2/28 patients; 7%, *p* = 0.03). Transmural necrosis on pathology was found in 2/7 resected patients with RI and 1/2 patients without RI and resected. The remaining 6/9 patients had limited mucosal or submucosal necrosis. Mean resected bowel length was 80.0 cm (46–115) in patients with RI and 48 cm (16–80) in patients without RI (*p* = 0.33).

**Fig. 5:**
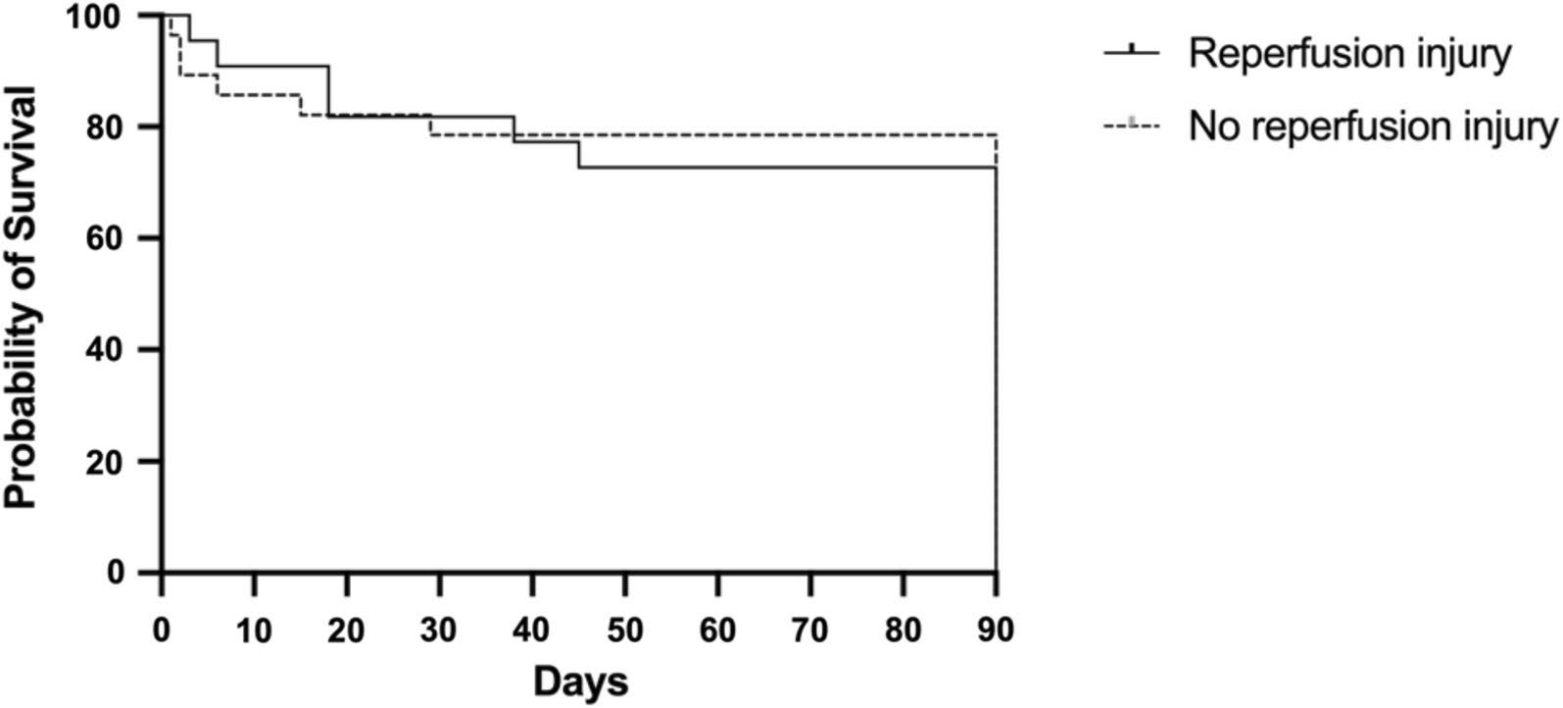
Kaplan–Meier curve for the three-month survival rate according to the occurrence of a reperfusion injury (log rank: *p* = 0.73)

The three-month survival rate of the cohort was 76%, with 12 deaths (all in-hospital), with no difference according to the occurrence of RI (22/28 vs. 16/22; log rank = 0.73); Fig. 5).

In the 22 patients with RI, the median follow-up was 354 days (66–989). After one-year follow-up, 11/22 patients were alive (61%) and 4/22 were lost to follow-up. In the 11/22 alive patients, five patients had a normal bowel on CT, one patient had a persistent and isolated jejunal thickening on CT and five did not undergo a follow-up CT.

## Discussion

This is the first study to evaluate RI in occlusive AAMI in humans. In this selected patient cohort, around 45% of the patients experienced RI, especially those who displayed a decreased bowel enhancement, a complete occlusion of the SMA on pre-treatment CT, in whom the cause was embolic and who presented with higher initial lactate serum levels. RI was also favored by an embolic cause, complete occlusion of the SMA, and higher initial lactate serum levels.

Despite a substantial amount of preclinical data assessing the biological or pathological process of bowel RI [19, 20], no clinical studies have been performed to date. Unlike in the field of myocardial [40] or cerebral ischemia [41], this significant gap between fundamental research and clinical practice is common in the field of mesenteric ischemia. Of course, AAMI is rare compared to ischemia in other organs [42], but more probably, this can be explained by the focus on bowel resection in historical surgical series [43]. As a result of improved understanding of the time-dependent pathophysiology of AAMI and identification of early forms of ischemia (i.e., with low probability of necrosis) that can benefit from endovascular revascularization, more recent studies discuss the primary importance of revascularization in clinical practice and research [2, 14, 44, 45]. This explains why the study population included mostly patients with a low risk of necrosis (i.e., Clichy score 0 or 1) that were referred by the on-call expert group to first-line endovascular revascularization [37].

Symptoms (abdominal pain and bloody diarrhea) after revascularization may be considered signs of persistent ischemia (incomplete treatment). However, they may also be due to reperfusion injury. Interestingly, the lactate and WBC were not discriminant, but the CRP level significantly increased after treatment in patients with RI, possibly due to bacterial translocation, as described in preclinical ischemia–reperfusion models [22]. These different findings show that once persistent AAMI has been excluded, reperfusion injury should be considered following an endovascular procedure and may mimic ischemia per se. Patients with RI presented with bowel wall thickening (nearly 9 mm in most patients) on imaging with wall hypoattenuation and mucosal hyperenhancement and fat stranding in more than 90% of cases. This pattern is different from that observed in AAMI and should be recognized to prevent diagnostic errors and unnecessary interventions.

Reliable prediction of RI in cases of AAMI potentially will potentially improves patient management. We identified several factors that could be helpful in clinical practice. Decreased bowel wall enhancement on pre-treatment CT was the strongest predictive factor (hazard radio > 8.0). The diagnostic value of this finding has been well described in occlusive AAMI [46–51], although the inter-observer variability is only fair to substantial [48] and its significance is a subject of debate [50] probably because most series include populations with both arterial and venous causes of ischemia [37, 48, 50]. We hypothesize that this feature is a sign of a deeper or more severe stage of ischemia in occlusive AAMI, leading to more extensive tissue and cellular lesions, explaining the increased frequency of RI. This is supported by the higher levels of serum lactate and the trend toward a higher Clichy score (i.e., 2 or 3) in the subgroup of patients who develop RI. Other predictive factors may also suggest more severe ischemia. Complete occlusion of the SMA, especially of an embolic origin, offers less perspective for the development of collateral anastomosis due to sudden clotting. These patients are usually treated by thrombus-aspiration and intra-arterial thrombolysis techniques rather than stenting [12, 44]. A shorter delay between the initial CT and revascularization was found in patients with RI but only in univariate analysis. This could be explained by the fact that these patients may experience sudden abdominal and rapidly morphine-resistant pain (evocative of an embolic origin). The time to diagnosis may therefore be shorter than in patient with atherosclerosis who may present with more progressive—however, acute—symptoms.

RI did not negatively influence patients’ short-term survival. However, the rate of secondary bowel resection was higher in patients with RI. This could be for two reasons. First, as discussed above, ischemia was more severe in patients with initial RI. Second, the clinical presentation (i.e., abdominal pain associated with increased CRP) may lead to more frequent surgical exploration and thus bowel resection. This shows the difficulty of managing RI, and the need to raise awareness about this event, which is underestimated and still poorly understood in clinical practice [28]. Thus, we believe that laparotomy should only be performed if additional features suggesting persistent ischemia are present, such as clear bowel hypo-enhancement, increased serum lactate levels, and of course, any organ failure. Furthermore, bowel dilatation, a key feature when assessing the probability of necrosis in AAMI [37, 52], may not be helpful after revascularization because ileus can occur in patients with RI alone and no necrosis.

Our study has certain limitations. First, the study design was retrospective, although it was based on a prospective institutional database. Moreover, the study cohort was relatively small due to the stringent inclusion and exclusion criteria (endovascular revascularization only, no surgery, CT within 7 days of treatment) to exclude any possible confounders.

The absence of systematic follow-up CT can represent a bias. However, nearly half of the follow-up CT (24/50) were performed with the objective to evaluate the quality of the revascularization even in patients with normal clinical and biological parameters. We can hypothesize that these patients had a low probability of necrosis and consequently a low probability of RI (because RI patients had a higher Clichy score). Furthermore, the number of patients excluded from the analysis because of no follow-up CT is relatively low (*n* = 12). Thus, the risk of underestimating RI remains low.

Our definition of angiographic technical success (that accounted for 92% of patients) may be discussed. It was defined as a complete or partial revascularization on the final angiogram which was purely arbitrary. Partial revascularizations may present as a large spectrum, from residual significant stenosis of the distal SMA to an occlusion of the third jejunal artery. However, technical success was not significantly associated with the occurrence of a reperfusion injury. Technical failure accounted in four patients and absent revascularization at control CT in eight patients. Therefore, four patients with technical success had a rethrombosis between the final angiogram and the control CT. This unfavorable evolution can occur and can be explained by multiple causes such as prothrombotic states (caused by systemic inflammation from AAMI), SMA intimal lesions (cause by thrombus-aspiration, for example), or insufficient anticoagulation/anti-platelet therapy.

Finally, the study did not include a validation cohort; thus, the present results require further validation.

In conclusion, RI is frequent following endovascular revascularization of occlusive AAMI. It is more frequent in patients with decreased bowel wall enhancement on pre-treatment CT, an embolic cause, and complete occlusion of the SMA, all features suggesting more severe ischemia. The rate of RI increases with the number of predictive factors. Nevertheless, the occurrence of RI does not impair short-term survival. Therefore, physicians and radiologists should be more aware of this entity to avoid unnecessary laparotomy and bowel resection.

## Supplementary Information

**Additional file 1:** Table. CT protocol for the assessment of mesenteric ischemia.

## Data Availability

The datasets used and/or analyzed during the current study are available from the corresponding author on reasonable request.

## Abbreviations

AAMI: Arterial acute mesenteric ischemia
CRP: C-reactive protein
CT: Computed tomography
HR: Hazard ratio
RI: Reperfusion injury
SMA: Superior mesenteric artery
WBC: White blood cells
