# Supplementary material for: Reperfusion injury on computed tomography following endovascular revascularization of acute mesenteric ischemia: prevalence, risk factors, and patient outcome
Source: Insights Imaging. 2022 Dec 13;13:194. doi: 10.1186/s13244-022-01339-9 (PMC9748024; doi:10.1186/s13244-022-01339-9)
Supplement: Supplementary file 1 — Additional file 1: Table. CT protocol for the assessment of mesenteric ischemia. [file 13244_2022_1339_MOESM1_ESM.pdf]

ELECTRONIC SUPPLEMENTARY MATERIAL

Reperfusion injury on computed tomography following endovascular revascularization of acute mesenteric ischemia: prevalence, risk factors, and patient outcome

Table. CT protocol for the assessment of mesenteric ischemia.

	**Non contrast phase**	**Arterial phase**	**Portal-venous phase**
Position	Supine	Supine	Supine
Coverage	Liver dome to pubic symphysis	Liver dome to pubic symphysis	Liver dome to pubic symphysis
Peak beam ernergy	120 kv	120 kv	120kv
mA mode	Modulated	Modulated	Modulated
Pitch	1.375	0.984	0.984
Slice Thickness	2.5 mm	1.25 mm	1.25 mm
Image reconstruction overlap	1.25 x 1 mm	1.25 x 1 mm	1.25 x 1 mm
FOV	Large	Large	Large
Delay acquisition		Bolus-triggered (beginning of the abdominal aorta) at 120 HU threshold	50 sec after arterial phase
Contrast media dose		2 mL/kg	
Contrast media Flow rate		4 mL/sec	
Iodine concentration		350 mg/mL	

Abbreviations: CT (Computed Tomography); FOV (Field-of-View)
